# Prevalence, Risk Factors, and Coinfection of Urogenital Schistosomiasis and Soil-Transmitted Helminthiasis among Primary School Children in Biase, Southern Nigeria

**DOI:** 10.1155/2021/6618394

**Published:** 2021-03-13

**Authors:** Kenneth Nnamdi Opara, Eteye Udobong Wilson, Clement Ameh Yaro, Luay Alkazmi, Nsima Ibanga Udoidung, Friday Maduka Chikezie, Bassey Eyibio Bassey, Gaber El-Saber Batiha

**Affiliations:** ^1^Department of Animal and Environmental Biology, University of Uyo, Akwa Ibom State, Nigeria; ^2^Biology Department, Faculty of Applied Sciences, Umm Al-Qura University, Makkah 21955, Saudi Arabia; ^3^Department of Pharmacology and Therapeutics, Faculty of Veterinary Medicine, Damanhour University, Damanhour 22511, Al Beheira, Egypt

## Abstract

Schistosomiasis and soil-transmitted helminthiasis (STH) are neglected tropical diseases (NTDs) that cause chronic infections and ill health. The study was carried out to determine the current infection status and risk factors associated with *Schistosoma haematobium* and soil-transmitted helminth (STH) coinfection among school children in Biase Local Government Area (LGA), Cross River State, Nigeria. A cross-sectional study was carried out. Urine and fecal samples were randomly collected from 630 school children in six villages of Biase LGA. Urine sedimentation and Kato-Katz techniques were used to diagnose urogenital schistosomiasis and STHs, respectively. A structured questionnaire was used to collect demographic information and risk factors. The prevalence of *S. haematobium* in Biase LGA was 6.03%, with males (27 pupils, 9.00%) significantly more (*χ*^2^ = 8.903, *p* value = 0.003, C.I. = −82.650–120.650) infected than the females (11 pupils, 3.33%), while the prevalence of STH infection was 11.27% with no significant difference (*χ*^2^ = 0.002, *p* value = 0.962, C.I. = −16.441–54.559) in prevalence between males (34 pupils, 11.33%) and females (37 pupils, 11.21%). Prevalence of *S. haematobium* and STHs ranged from 1.82 to 19.13% and from 4.55 to 19.05% within the communities, respectively, with Abini (22 pupils, 19.13%) and Adim (20 pupils, 19.05%) communities having the highest prevalence for *S. haematobium* and STHs, respectively. The most infected age group was 11–13 years (21 pupils, 9.68%) for *S. haematobium* and 14–16 years (5 pupils, 21.74%) for STHs. *Ascaris lumbricoides*, hookworms, and *Trichuris trichiura* had prevalence of 5.56%, 3.02%, and 2.70%, respectively. An overall prevalence of 7.14% and 8.41% was observed for haematuria and proteinuria, respectively. Prevalence of coinfection among the parasites was 4.76%. Male pupils (OR = 2.868, C.I.: 1.397–5.889), pupils of the age group of 11–13 years (OR = 2.496, C.I.: 1.287–4.838), school children that swim (OR = 1.527, C.I.: 0.784–2.974), those that cross streams to farm (OR = 25.286, C.I.: 4.091–156.283), those that visit stream or river severally (OR = 3.077, C.I.: 1.204–7.863), and those whose home is 1 km (OR = 3.116, C.I.: 1.292–7.518) from the stream are at higher odds of infection with *S. haematobium*. For STHs, male pupils (OR = 1.012, C.I.: 0.617–1.659), pupils of the age group of 11–13 years (OR = 2.609, C.I.: 1.582–4.302), pupils that walk barefoot (OR = 18.746, C.I.: 6.786–51.783), those that do not wash fruits and vegetables before eating (OR = 2.334, C.I.: 1.400–3.892), those that do not wash hands after using the toilet (OR = 1.200, C.I.: 0.730–1.973), those that eat soils (OR = 2.741, C.I.: 1.533–4.902), those that drink water from streams or rivers (OR = 189.509, C.I.: 24.807–1447.740), and those that use pit latrine (OR = 2.920, C.I.: 1.746–4.885) and/or open defecation (OR = 2.552, C.I.: 1.454–4.479) are at high odds of being infected with STHs. Urogenital schistosomiasis and soil-transmitted helminthiasis are still endemic diseases in Biase LGA. Although the degree of infection is quite low or moderate, there is a need to intensify and sustain control measures such as provision of sustainable clean water supply, health education intervention, and chemotherapy.

## 1. Background

Schistosomiasis and soil-transmitted helminthiasis are classified by the World Health Organization (WHO) as neglected tropical diseases (NTDs) [1] and are among the most prevalent NTDs [2]. Soil-transmitted helminth (STH) infections are endemic in 166 countries worldwide [3] while schistosome infections are endemic in 76 countries [4] with a global combined burden of more than 3 billion people occurring mainly in sub-Saharan Africa, South and North America, China, and East Asia [5]. These infections mainly affect the poorest and most deprived communities which are characterized by poor sanitation, lack of potable water, inadequate health facilities, poor housing, overcrowding, and squalid environment [6–8].

Nigeria has been identified as the country with the highest burden of schistosomiasis and soil-transmitted helminthiasis (STH) in sub-Saharan Africa. Nigeria also has the greatest number of cases of schistosomiasis worldwide. The most important species causing schistosomiasis are *Schistosoma haematobium* and *Schistosoma mansoni* [9, 10]. Urogenital schistosomiasis is caused by *Schistosoma haematobium* while intestinal schistosomiasis is caused by *S. mansoni.* Soil-transmitted helminthiasis is caused by ingestion of eggs of *Ascaris lumbricoides* and *Trichuris trichiura* or by active penetration of the skin by larvae of *Ancylostoma duodenale* and *Necator americanus* (hookworms) in the soil [11, 12]. Infections with these parasites are known to be associated with hepatosplenomegaly and portal hypertension, chronic inflammation, gastrointestinal problems, anaemia, and depletion of nutrients in children, thereby leading to adverse effect on physical and cognitive development [13–15]. School-aged children (SAC) harbour the highest prevalence and intensity of schistosome and STH infections in sub-Saharan Africa (SSA) [9].

Currently, control measures adopted in Nigeria consist of treatment once annually with either albendazole or mebendazole for STH infections and praziquantel for schistosome infections. This programme is achieved through school-based deworming (SBD) carried out by the State Ministries of Health in collaboration with the Federal Ministry of Health Nigeria (FMoH), WHO, and other nongovernmental organizations (NGOs). This programme [16] offers treatment of all school children in the country. Due to the poor environment as well as poor hygiene behaviour by individuals, reinfection occurs rapidly after treatment. Therefore, there is a need for constant surveillance to determine the current infection status and geographical overlap between soil-transmitted helminths and schistosomes. This study investigated the current status of *S. haematobium* and soil-transmitted helminth coinfection and associated risk factors in Biase Local Government Area (LGA), Cross River State, Nigeria.

## 2. Methods

### 2.1. Study Area

**Figure 1:**
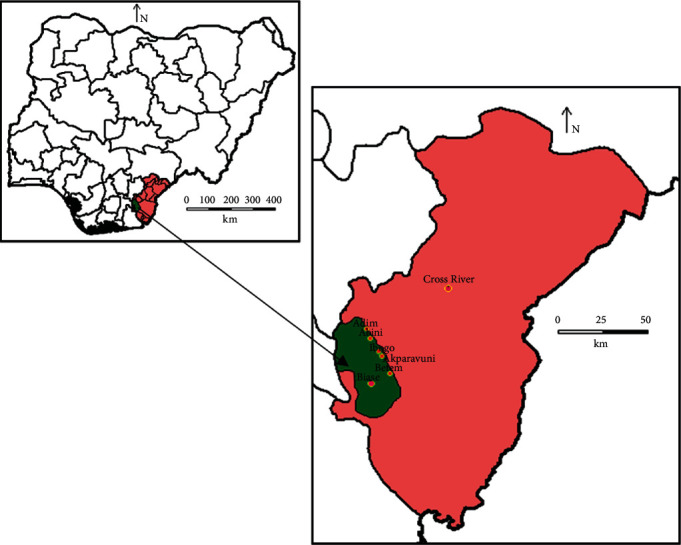
Map of Biase Local Government Area showing sampling communities. Source: Department of Geography, University of Uyo, Nigeria.

The study was conducted in Biase LGA, Cross River State, Nigeria. It has an area of 1,310 km^2^. The LGA lies on latitude 5°32′N and 4°27′N and longitude 7°50′E and 9°28′E. Cross River State shares boundaries with the Republic of Cameroon in the east, Benue State in the north, Ebonyi and Abia states in the west, Akwa lbom State in the southwest, and the Atlantic Ocean in the south. It has a total landmass of about 23,000 sqkm (Figure 1). The state has a 2015 projected population of 3,783,085 people [17].

The climate is tropical except for Obudu Plateau which has an altitude of 1,575.76 m above the sea level with a temperate climate. All-year-round rainfall of about 350 mm occurs along the coastal area. Rainfall in the hinterland is between 120 and 200 mm annually with maximum precipitation occurring from July to September. Ambient temperatures remain high throughout the year (22.4-33.2°C). Relative humidity is high (60-93%). The people of Biase are predominantly farmers, and the tropical environment coupled with the rich soils supports the cultivation of swamp rice.

### 2.2. Study Design

The study was carried out from October 2017 to January 2018. This cross-sectional survey involves sample collection from school-aged children aged between 5 and 16 years from six (6) purposively selected communities in Biase LGA. In each of the selected community, a simple random sampling was used to select a school.

### 2.3. Ethical Considerations

Ethical permission for the study was obtained from the ethical board of Cross River State Ministry of Health, Calabar, with reference number: CRSMOH/RP/REC/2017/524. Advocacy visits were made to the Biase Local Government prior to the commencement of the study. Full informed consent was obtained from parents/guardians. Approval to conduct the study in the schools was obtained from the Education Secretary, Biase Local Education Authority; this approval was communicated to the head teachers of schools before sample collection.

### 2.4. Study Population

The study population includes school-aged children of 5-16 years in primary schools in Biase Local Government Area, Cross River State, Nigeria.

### 2.5. Inclusion and Exclusion Criteria

Only school children whose guardians and caregivers signed the consent form participated in the study. Children who refused to participate or whose parents/guardians did not give consent, menstruating school girls, children who were absent from school, and those who were unable to produce urine and fecal samples were excluded from the exercise.

### 2.6. Sample Size Determination

This was estimated using the sample size formula [18] as calculated below:

(1) $$\begin{array}{c} n=\frac{Z^{2}\times p\left(1-p\right)}{e^{2}}, \end{array}$$

*Z* = 1.96 (95%), *p* = prevalence of urogenital schistosomiasis = 50%, and *e* = error rate 0.05 (5%),

(2) $$\begin{array}{c} n=\frac{1.96^{2}\times 0.5\left(1-0.5\right)}{0.05^{2}}, \end{array}$$

*n*≃384 + 20%nonrespondent value = 384 + 77 = 461.

The minimum sample size is 461 pupils. A total of 630 school children, comprising of 300 males and 330 females, were selected randomly from the surveyed schools.

### 2.7. Sample Collection

A total of 630 school children from six primary schools were randomly selected for the study. All the pupils eligible for the study were lined up according to class by sex, and selection was done randomly by selecting a sampling frame at equal intervals depending on the total number of children who were included in the study from each school.

School children were given sterile screw-capped plastic containers, well labelled with age and sex, to collect their urine samples between 10a.m. and 2p.m., a period when peak excretion of eggs is expected [19]. School children were also given sterile capped plastic containers (well labelled with age and sex) to collect 10 g of fresh fecal specimens. The collected samples were transported, for laboratory analysis as described by WHO [20, 21].

### 2.8. Questionnaire Administration

Structured questionnaires were administered to solicit information on sociodemographic and socioeconomic factors such as age, sex, parent's occupation and education, type of house, and personal hygiene practices of the participants. Information was gathered with the aid of the class teachers in the various schools. Local dialect was used to communicate with children who do not understand English.

### 2.9. Parasitological Examination

Stool samples collected were examined within 24 hours and examined in duplicate for the presence of STH parasites using the Kato-Katz thick smear technique as described by WHO [22]. This involved straining stool through the Kato-Katz sieve with a mesh size of 250 *μ*m. The fine stool was filled in a Kato-Katz template producing 41.7 mg. Using the measured fine stool, a Kato-Katz thick smear was prepared on a slide, and this was covered with cellophane coverslips soaked in 50% glycerine-malachite green. The slides were examined, and all identified eggs were recorded [14].

A sedimentation quantitative technique was employed for the detection of *S. haematobium* eggs in the urine samples as described by Cheesbrough [21]. Briefly, 10 ml of each thoroughly mixed urine sample was put in a centrifuge tube. The centrifuge was spun for 5 minutes at 1500 rpm. The supernatant was decanted leaving about 0.5 ml of the fluid with the sediment at the bottom of the tube undisturbed. The remaining fluid and sediment were mixed; then, a drop of the mixture was transferred to a microscope slide and covered with a coverslip. The slide was meticulously examined for *S. haematobium* using ×10 objectives [14].

### 2.10. Examination of Urine for Haematuria and Proteinuria

Within 2 hours of collection of the urine samples, haematuria and proteinuria were detected in the field using dipsticks (Medi-Test Combi 9 manufactured by Machery-Nagel Duren, Germany). The reagent end of the test strip was dipped into fresh, well-mixed uncentrifuged urine for 40 seconds. Upon removal, the test area was compared with a standard colour as described by the manufacturer [20, 23].

### 2.11. Statistical Analyses

Data obtained were entered into “Microsoft Excel” for Windows, version 2013 (Microsoft Corporation, Redmond, Washington, USA). Descriptive statistics were used to compute the prevalence and confidence interval (CI) of the study population. A chi-squared test was used to determine the relationship in the prevalence of STHs according to sex, age groups, and communities. Bivariate and multivariate analyses were used to determine the level of association of risk factors with infection status; the odds ratio (OR) was determined. All analyses were performed using the Statistical Package for the Social Sciences (SPSS) software for Windows, version 20.0 (SPSS Inc., Chicago, IL, USA).

## 3. Results

### 3.1. Demographic Characteristics

A total of 630 pupils were examined for *S. haematobium* and STHs in Biase LGA comprising of males (300 pupils, 47.52%) and females (330 pupils, 52.38%). According to the age distribution of school children examined, majority fell within the age group of 8–10 years (21 pupils, 50.92%), followed by the age group of 11–13 years (217 pupils, 34.40%) and 5–7 years (69 pupils, 10.96%) while the age group of 14–16 years (23 pupils, 3.70%) had the least number of children examined (Table 1).

### 3.2. Prevalence of *S. haematobium*

School children from Biase LGA were observed to be infected with *S. haematobium* (38 pupils, 6.03%) with females (27 pupils, 9.00%) having a higher prevalence than males (11 pupils, 3.33%); a significant difference (*χ*^2^ = 8.903, *p* value = 0.003, C.I. = −82.650–120.650) exists between the sexes in the prevalence of *S. haematobium*. According to the communities, Abini (22 pupils, 19.13%) had the highest prevalence of *S. haematobium*, followed by Adim (12 pupils, 11.43%) and Ibogo (2 pupils, 2.22%), while Akpet community (2 pupils, 1.82%) had the least prevalence (Table 2); school children examined from Akparavuni and Betem were not infected with *S. haematobium*. A comparison of the prevalence according to communities revealed significant differences (*χ*^2^ = 59.437, *p* value < 0.001, C.I. = −2.998–15.665). According to the age group, school children within the age group of 11–13 years (21 pupils, 9.68%) had the highest prevalence while the age group of 8–10 years (13 pupils, 4.10%) had the least prevalence. There is no significant difference (*χ*^2^ = 8.903, *p* value = 0.051, C.I. = −5.285–24.285) in prevalence according to age groups (Table 2).

### 3.3. Prevalence of Haematuria and Proteinuria

Haematuria and proteinuria were observed to be highest in pupils from Abini (haematuria: 24 pupils, 20.87%; proteinuria: 26 pupils, 22.61%) and Adim (haematuria: 12 pupils, 11.43%; proteinuria: 11 pupils, 10.48%) communities. Haematuria (*χ*^2^ = 51.213, *p* value < 0.001, C.I. = −1.951–16.951) and proteinuria (*χ*^2^ = 42.689, *p* value < 0.001, C.I. = −0.610–19.276) were significantly different among the communities (Table 2). According to the age group, children within the age group of 11–13 years had the highest haematuria (26 pupils, 11.98%) and proteinuria (24 pupils, 11.06%); haematuria (*χ*^2^ = 19.178, *p* value < 0.001, C.I. = −6.296–28.796) and proteinuria (*χ*^2^ = 16.279, *p* value < 0.001, C.I. = −5.003–31.503) differ significantly among the age groups. The prevalence of haematuria (male: 30 pupils, 10.00%; female: 15 pupils, 4.55%; *χ*^2^ = 7.949, *p* value 0.008, C.I.:−72.797–117.797) and proteinuria (male: 34 pupils, 11.33%; female: 19 pupils, 5.76%; *χ*^2^ = 6.341, *p* value 0.012, C.I.: 6.341–121.797) differs significantly between the sexes with males having higher prevalence than females (Table 2).

### 3.4. Prevalence of STHs

The overall prevalence of STHs in the study area was 11.27% with males (34 pupils, 11.33%) having higher prevalence than females (37 pupils, 11.21%); there is no significant difference (*χ*^2^ = 0.002, *p* value =0.962, C.I. = −16.441–54.559) between sexes in STH prevalence (Table 2). Prevalence varied among the communities with Adim (20 pupils, 19.05%) having the highest, followed by Betem (18 pupils, 16.36%), Abini (12 pupils, 10.44%), Ibogo (9 pupils, 10.00%), and Akparavuni (7 pupils, 7.00%) while Akpet Central (5 pupils, 4.55%) had the least prevalence; a significant difference (*χ*^2^ = 16.229, *p* value < 0.001, C.I. = 5.487–18.179) was observed in the prevalence among the communities. The age group of 14-16 years (5 pupils, 21.74%) had the highest infection with STHs, followed by the age group of 11–13 years (39 pupils, 17.97%) and 8–10 years (24 pupils, 7.48%) while the age group of 5–7 years (3 pupils, 4.35%) had the least prevalence. A comparison of the prevalence among the age groups revealed a significant difference (*χ*^2^ = 20.195, *p* value < 0.001, C.I. = −9.359–44.859) (Table 2).

The prevalence of STH parasites observed was low. *A. lumbricoides* (35 pupils, 5.56%) had the highest prevalence, followed by hookworms (19 pupils, 3.02%) while *T. trichiura* (17 pupils, 2.70%) had the least prevalence. A comparison of the prevalence of *A. lumbricoides* (*χ*^2^ = 26.300, *p* value < 0.001, C.I. = 0.069–11.597) and hookworms (*χ*^2^ = 23.419, *p* value < 0.001, C.I. = −0.839–7.172) among the communities revealed a significant difference (Table 3).

### 3.5. Coinfection of *S. haematobium* and STHs

Of the 630 pupils examined for parasites, 4.76% were observed to harbour multiple parasites. Hookworms and *T. trichiura* were coinfected than other parasites with a prevalence of 1.6%; their coinfection significantly differs (*χ*^2^ = 13.022, *p* value = 0.043) from that of other parasites (Table 4).

### 3.6. Risk Factors of Urogenital Schistosomiasis and Soil-Transmitted Helminthiasis

Analysis of the risk factors associated with Urogenital Schistosomiasis is presented in Table 5. The multivariate analysis of risk factors revealed the groups associated with high odds of infection. Males (OR = 2.868, C.I.: 1.397–5.889) have 3 times higher odds of being infected than females. School children within the age group of 11–13 years (OR = 2.496, C.I.: 1.287–4.838) were significantly associated with infection with *S. haematobium* 2 1/2 times higher than children in other age groups. School children whose fathers are daily labourers (OR = 64.667, C.I.: 26.494–157.840) and whose mothers' are either farmers (OR = 2.963, C.I.: 1.486–5.909) or traders (OR = 2.733, C.I.: 0.768–9.723) have higher odds of infections with *S. haematobium*. According to educational status of the parents, a child whose father (OR = 90.615, C.I.: 24.092–340.824) and mother (OR = 6.748, C.I.: 2.270–20.060) had no formal education has higher odds of being infected than children whose parents are educated. School children that swim (OR = 1.527, C.I.: 0.784–2.974) and cross streams to farm (OR = 25.286, C.I.: 4.091–156.283) have higher odds of being infected with *S. haematobium*. School children who visit stream or river twice (OR = 1.949, C.I.: 1.001–3.771) or severally (OR = 3.077, C.I.: 1.204–7.863) are 2 times or 3 times likely to be infected than those that visit the stream once (Table 5). Children whose home is 1 km (OR = 3.116, C.I.: 1.292–7.518) from the stream are at higher odds of infection than those of 2 km and beyond. Also, school children with no knowledge about schistosomiasis (OR = 3.739, C.I.: 1.740–8.038) are 4 times likely to be infected than those with knowledge about schistosomiasis.

For STHs, the multivariate analysis revealed that males (OR = 1.012, C.I.: 0.617–1.659) have higher odds of being infected with STHs than females. The age groups of 11–13 years (OR = 2.609, C.I.: 1.582–4.302) and 14–16 years (OR = 2.277, C.I.: 0.818–6.335) are associated with STH infections. Children whose fathers are daily labourers (OR = 4.418, C.I.: 1.977–9.872) are likely to get infected 4 1/2 times than those whose parents are into other occupations (Table 6). Only children whose mothers' are civil servants have low risk of infections with STHs compared to those whose parents are into farming, trading, housewife, and other occupations. Children whose fathers (OR = 37.695, C.I.: 10.342–137.394) and mothers (OR = 24.835, C.I.: 8.550–72.135) have no formal education are 38 times and 25 times likely to be infected with STHs than those whose parents are educated. Pupils that walk barefoot (OR = 18.746, C.I.: 6.786–51.783), do not wash fruits and vegetables before eating (OR = 2.334, C.I.: 1.400–3.892), do not wash hands after using toilet (OR = 1.200, C.I.: 0.730–1.973), and eat soils (OR = 2.741, C.I.: 1.533–4.902) have higher odds of being infected with STHs and are significantly associated with infections from STHs. School children that drink water from streams or rivers (OR = 189.509, C.I.: 24.807–1447.740) and those that use pit latrine (OR = 2.920, C.I.: 1.746–4.885) and/or open defecation (OR = 2.552, C.I.: 1.454–4.479) are at high odds of being infected with STHs. School children that were never dewormed (OR = 41.388, C.I.: 21.552–79.477) are significantly associated with high infection from STHs (Table 6).

## 4. Discussion

This study revealed that *S. haematobium* and STHs are still endemic in Biase, Cross River State, Nigeria. Schistosomiasis and soil-transmitted helminthiasis are among the NTDs which occur predominantly due to inadequate basic amenities and poor environmental hygiene in rural areas [22]. The prevalence of urogenital schistosomiasis and soil-transmitted helminthiasis in this study is lower when compared to the observation of a study in Akpet community, Cross River State [24], where 19% and 20% prevalence was observed for urogenital schistosomiasis and intestinal parasites, respectively. The prevalence of these parasites was also lower than the prevalence of 62.2% and 32.7% observed in school children in Sarki, Oyo State, Nigeria [25], for *S. haematobium* and soil-transmitted helminths, respectively. In the rural areas of KwaZulu-Natal, South Africa [26], prevalence of 36.8% and 38.8% was observed for *S. haematobium* and STHs, respectively. The higher prevalence in previous studies and the variation in prevalence among the communities in this study might be due to differences in environmental conditions, cultural practices, inequality in safe water resources, sanitation, and personal hygiene of the pupils. Also, mass drug administration of school children carried out previously by the State Ministry of Health might be responsible for the low prevalence.

School-aged children have been shown to be at greater risk of schistosomiasis and STHs [27–30]. The highest prevalence of *S. haematobium* observed in this study within the age group of 11–13 years agreed with a study carried out in Lafia, Nasarawa State, Nigeria [31] with the highest prevalence recorded within the age group 11–15 years. Also, the prevalence of STHs in this study increases drastically in the age group of 11–13 years and 14–16 years. The prevalence of 21.74% observed in the age group 14-16 years is higher than 11.8% reported by Odinaka et al. [32] in the same age group in Imo State, Nigeria. The high prevalence noted in the age groups of 11–13 years and 14–16 years might be due to their being the most active age groups that are involved in daily outdoor activities such as fetching water from streams and rivers, swimming in infested water bodies, and playing barefooted. Also, school children were also observed to eat indiscriminately with unwashed hands.

A higher prevalence of urogenital schistosomiasis was observed in males than females which was statistically significant, indicating that males were more exposed to the infection than females through water contacts. The high prevalence observed in males agreed with previous studies conducted in Senegal [33] and Ghana [34]. The reason was that the males were more likely to be knowledgeable of their environments including water bodies and were more likely to play in them compared to the females. The gender role in the society could as well explain why males were more likely to engage in open water contact practices than females [35].

Out of the 45 school children that were positive for haematuria, only 38 (84.44%) had *S. haematobium* eggs in their urine. Not all cases of haematuria tested positive with *Schistosoma* eggs, and this result corresponds with earlier reports [36]. The most common and visible sign of *S. haematobium* infection is haematuria while further analysis may also show proteinuria. From this study, it was observed that most of the children who self-reported haematuria also tested positive for *S. haematobium* infection and had proteinuria. This suggests that haematuria and proteinuria might be a diagnostic indicator of infection with *S. haematobium*. A similar result has been reported in Ghana [34] and Senegal [37].

In the present study, the children were mostly infected with STH than schistosomiasis, an observation that is consistent with the findings of a study carried out in Northeastern Mindanao [36]. All STHs combined were more prevalent than *S. haematobium*. This findings however contradicts the report of a study in Cameroon [38] that observed a high prevalence of schistosomiasis than STHs. The most prevalent STHs in this study was *A. lumbricoides*. In this study, coinfections between urogenital schistosomiasis and STHs were low. This is in consonance with studies carried out in Northeastern Mindanao [36] and in Ogun State, Nigeria [39], with prevalence of 6.4% and 1.4%, respectively, but different from an earlier observation in Akpet community, Cross River State, Nigeria [24], that reported a prevalence of 10.6%. The low prevalence of coinfections indicated that *S. haematobium* and STHs were independently distributed among study participants. This might be due to the fact that the transmission dynamics of *S. haematobium* and soil-transmitted helminths were different. The coinfection observed in this study could be as a result of overlap of the conditions that favor coexistence of both urogenital schistosomiasis and STH. Some of these conditions include the existence of wet soils maintained by high rainfalls, optimal temperatures for parasite survival, and domestic activities that predispose communities to infections. These activities include bathing and washing clothes in rivers and streams thereby increasing the frequency of exposure of people to contaminated water bodies and poor sanitation facilities that result in environmental contamination with feces [6].

Sex (male), age group (11–13 years), water contact practices (swimming and crossing water to farms), frequency of visitation to streams (twice and severally), and distance of home from the stream (1 km) were significantly (*p* < 0.05) associated with urogenital schistosomiasis. This agreed with the studies elsewhere [34, 40]. Meanwhile, playing barefoot, source of drinking water, open defecation, not washing hands after using the toilet, eating of soils, and treatment for worms were significantly (*p* < 0.05) associated with STHs. Despite hygiene education given as part of the deworming campaign in Biase, a large number of school children still walk barefooted, do not wash hands after toilet, and still eat soil.

The low overall prevalence of *S. haematobium* and STHs observed in this study could be attributed to the integrated control measures put in place through the annual administration of praziquantel and albendazole to school-aged children by the Cross River State Ministry of Health. As preventive chemotherapy does not guard against reinfection, broadening the scope of intensified intervention against schistosomiasis and STH to go beyond that of current anthelminthic treatment regimens alone is crucial; hence, WASH-related interventions that reduce environmental transmission and exposure are important [41, 42].

This study has some limitations which might be of interest in interpreting the result. One concern is the limitation of local technicians in detecting helminth infection [36, 43]. Another is the diagnostic techniques adopted in this study, namely, Kato-Katz and sedimentation, which might have underestimated the true prevalence of STH and urogenital schistosomiasis. A more accurate prevalence would have been estimated if a more sensitive diagnostic method was used.

## 5. Conclusion

Urogenital schistosomiasis and soil-transmitted helminthiasis are still endemic in Biase Local Government Area, Cross River State. The age of pupils, sex, water contact activities, playing barefoot, source of drinking water, and open defecation are risk factors significantly associated with urogenital schistosomiasis and STHs in Biase. There is an urgent need to heighten control strategies in these communities to further reduce the burden of infection. Effective health education campaigns on preventive and control measures alongside chemotherapy should be carried out and sustained in schools.

## Figures and Tables

**Table 1:** Demographic characteristics of school children in Biase LGA, Cross River State.

Categories	Number examined	%
*Communities*		
Abini	115	18.25
Adim	105	16.70
Akparavuni	100	15.87
Akpet Central	110	17.46
Betem	110	17.46
Ibogo	90	14.28
*Age (years)*		
5–7	69	10.96
8–10	321	50.92
11–13	217	34.40
14–16	23	3.70
*Sex*		
Male	300	47.62
Female	330	52.38
Total	630	100.00

**Table 2:** Prevalence of *S. haematobium*, STHs, haematuria, and proteinuria.

Categories (*n*)	Number infected with *S. haematobium* (%)	Number infected with STHs (%)	Number with haematuria (%)	Number with proteinuria (%)
*Communities*				
Abini (115)	22 (19.13)	12 (10.44)	24 (20.87)	26 (22.61)
Adim (105)	12 (11.43)	20 (19.05)	12 (11.43)	11 (10.48)
Akparavuni (100)	0 (0.00)	7 (7.00)	2 (2.00)	3 (3.00)
Akpet Central (110)	2 (1.82)	5 (4.55)	1 (0.91)	7 (6.36)
Betem (110)	0 (0.00)	18 (16.36)	3 (2.73)	3 (2.73)
Ibogo (90)	2 (2.22)	9 (10.00)	3 (3.33)	3 (3.33)
Total (630)	38 (6.03)	71 (11.27)	45 (7.14)	53 (8.41)
*χ* ^2^	59.437	16.229	51.213	42.689
Df	5	5	5	5
*p* value	<0.001^∗^	<0.001^∗^	<0.001^∗^	<0.001^∗^
C.I.	-2.998–15.665	5.487–18.179	-1.951–16.951	-0.610–19.276
*Age (years)*				
5–7 (69)	3 (4.40)	3 (4.35)	5 (7.25)	6 (8.70)
8–10 (321)	13 (4.10)	24 (7.48)	13 (4.05)	22 (6.85)
11–13 (217)	21 (9.68)	39 (17.97)	26 (11.98)	24 (11.06)
14–16 (23)	1 (4.35)	5 (21.74)	1 (4.35)	1 (4.35)
Total (630)	38 (6.03)	71 (11.27)	45 (7.14)	53 (8.41)
*χ* ^2^	7.773	20.195	19.178	16.279
Df	3	3	3	3
*p* value	0.051	<0.001^∗^	<0.001^∗^	<0.001^∗^
C.I.	-5.285–24.285	-9.359–44.859	-6.296–28.796	-5.003–31.503
*Sex*				
Male (300)	27 (9.00)	34 (11.33)	30 (10.0)	34 (11.33)
Female (330)	11 (3.33)	37 (11.21)	15 (4.55)	19 (5.76)
Total (630)	38 (6.03)	71 (11.27)	45 (7.14)	53 (8.41)
*χ* ^2^	8.903	0.002	7.049	6.341
Df	1	1	1	1
*p* value	0.003^∗^	0.962	0.008^∗^	0.012^∗^
C.I.	-82.650–120.650	-16.441–54.559	-72.797–117.797	-68.797–121.797

*n*: number examined. ^∗^Significant at *p* ≤ 0.05.

**Table 3:** Prevalence of STHs according to communities in Biase LGA, Cross River State.

Communities (*n*)	*Ascaris lumbricoides* (%)	Hookworms (%)	*Trichuris trichiura* (%)	STHs (%)
Abini (115)	7 (6.09)	2 (1.74)	3 (2.61)	12 (10.44)
Adim (105)	16 (15.24)	0 (0.00)	4 (3.81)	20 (19.05)
Akparavuni (100)	2 (2.00)	0 (0.00)	5 (5.00)	7 (7.00)
Akpet Central (110)	3 (2.73)	2 (1.82)	0 (0.0)	5 (4.55)
Betem (110)	6 (5.46)	10 (9.09)	2 (1.82)	18 (16.36)
Ibogo (90)	1 (1.11)	5 (5.56)	3 (3.33)	9 (10.00)
Total (630)	35 (5.56)	19 (3.02)	17 (2.70)	71 (11.27)
*χ* ^2^	26.300	23.419	6.028	16.229
Df	5	5	5	5
*p* value	<0.001^∗^	<0.001^∗^	0.303	<0.001^∗^
C.I.	0.069–11.597	-0.839–7.172	1.026–4.641	5.487–18.179

*n*: number examined. ^∗^Significant at *p* ≤ 0.05.

**Table 4:** Prevalence of coinfection of STHs and *Schistosoma haematobium.*

Parasites	Number examined	Prevalence (%)
*A. lumbricoides*+hookworms	630	3 (0.48)
*A. lumbricoides*+*Trichuris trichiura*	630	5 (0.79)
Hookworms+*Trichuris trichiura*	630	10 (1.59)
*Ascaris lumbricoides*+*Schistosoma haematobium*	630	2 (0.32)
Hookworms+*Schistosoma haematobium*	630	6 (0.95)
*Trichuris trichiura*+*Schistosoma haematobium*	630	3 (0.48)
Triple infection	630	1 (0.16)
Total		30 (4.76)
*χ* ^2^		13.022
Df		6
*p* value		0.043^∗^

^∗^Significant at *p* ≤ 0.05.

**Table 5:** Bivariate and multivariate analysis of risk factors associated with urogenital schistosomiasis among school children in Biase LGA.

Factors (*n*)	Number positive (%)	*χ* ^2^	Df	*p* value	Adjusted odds ratio (95% C.I.)
*Sex*					
Male (300)	27 (9.00)	7.931	1	0.005^∗^	2.868 (1.397-5.889)^¥^
Female (330)	11 (3.33)	7.931	1	0.005^∗^	0.349 (0.170–0.716)
*Age group (years)*					
5–7 (69)	3 (4.35)	0.126	1	0.723	0.683 (0.204–2.283)
8–10 (321)	13 (4.05)	3.851	1	0.050^∗^	0.480 (0.241–0.955)
11–13 (217)	21 (9.68)	6.812	1	0.009^∗^	2.496 (1.287–4.838)^¥^
14–16 (23)	1 (4.35)	0.000	1	1.000	0.700 (0.092–5.340)
*Father's occupation*					
Farming (327)	9 (2.75)	11.726	1	<0.001^∗^	0.267 (0.124–0.575)
Trading (228)	5 (2.19)	8.259	1	0.004^∗^	0.251 (0.097–0.652)
Civil servant (31)	3 (9.68)	0.238	1	0.626	1.725 (0.500–5.958)
Daily labourer (30)	20 (66.67)	193.252	1	<0.001^∗^	64.667 (26.494–157.840)^¥^
Others (14)	1 (7.14)	0.000	1	1.000	1.204 (0.153–9.454)
*Mother's occupation*					
Civil servant (343)	10 (2.92)	11.722	1	<0.001^∗^	0.278 (0.133–0.582)
Farming (258)	25 (9.69)	9.252	1	0.002^∗^	2.963 (1.486–5.909)^¥^
Trading (21)	3 (14.29)	1.322	1	0.250	2.733 (0.768–9.723)
Housewife (2)	0 (0.00)	NA	NA	NA	NA
Others (6)	0 (0.00)	NA	NA	NA	NA
*Father's educational status*					
Primary (175)	10 (5.71)	0.000	1	0.983	0.924 (0.439–1.945)
Secondary (340)	8 (2.35)	16.255	1	<0.001^∗^	0.209 (0.094–0.463)
Tertiary (100)	8 (8.00)	0.452	1	0.501	1.449 (0.644–3.261)
No formal education (15)	12 (80.00)	135.261	1	<0.001^∗^	90.615 (24.092–340.824)^¥^
*Mother's educational status*					
Primary (293)	11 (3.75)	4.290	1	0.038	0.448 (0.218–0.920)
Secondary (292)	19 (6.51)	0.089	1	0.766	1.169 (0.606–2.252)
Tertiary (27)	3 (11.11)	0.518	1	0.472	2.029 (0.582–7.065)
No formal education (18)	5 (27.78)	11.762	1	<0.001^∗^	6.748 (2.270–20.060)^¥^
*Water contact activities*					
Bathing (174)	7 (4.02)	1.257	1	0.262	0.575 (0.248–1.331)
Swimming (207)	16 (7.73)	1.153	1	0.283	1.527 (0.784–2.974)
Cross streams to farm (5)	3 (60.00)	17.190	1	<0.001^∗^	25.286 (4.091–156.283)^¥^
Washing (98)	5 (5.10)	0.036	1	0.849	0.813 (0.309–2.137)
Fetching (146)	7 (4.79)	0.268	1	0.604	0.736 (0.317–1.708)
*Number of times visit the stream or river*					
Once (385)	14 (3.64)	8.945	1	0.003^∗^	0.348 (0.176–0.686)
Twice (205)	18 (8.78)	3.364	1	0.067	1.949 (1.001–3.771)^¥^
Severally (40)	6 (15.00)	4.489	1	0.034^∗^	3.077 (1.204–7.863)^¥^
*Distance of home from the stream*					
1 km (47)	7 (14.89)	5.449	1	0.020^∗^	3.116 (1.292–7.518)^¥^
2 km (325)	21 (6.46)	0.090	1	0.764	1.170 (0.605–2.263)
>3 km (258)	10 (3.88)	2.967	1	0.085	0.495 (0.236–1.039)
*Knowledge of schistosomiasis*					
Yes (327)	9 (2.75)	11.726	1	<0.001^∗^	0.267 (0.124–0.575)
No (303)	29 (9.57)	11.726	1	<0.001^∗^	3.739 (1.740–8.038)^¥^

^¥^Significantly associated with urogenital schistosomiasis; *n*: number examined; ^∗^significant at *p* < 0.05.

**Table 6:** Bivariate and multivariate analysis of risk factors associated with STHs among school children in Biase LGA.

Factors (*n*)	Number positive (%)	*χ* ^2^	Df	*p* value	Adjusted odds ratio (95% C.I.)
*Sex*					
Male (300)	34 (11.33)	0.000	1	1.000	1.012 (0.617–1.659)^¥^
Female (330)	37 (11.21)	0.000	1	1.000	0.988 (0.603–1.620)
*Age group (years)*					
5–7 (69)	3 (4.35)	2.976	1	0.085	0.330 (0.101–1.077)
8–10 (321)	24 (7.48)	8.660	1	<0.001	0.451 (0.268–0.757)
11–13 (217)	39 (17.97)	13.866	1	<0.001	2.609 (1.582–4.302)^¥^
14–16 (23)	5 (21.74)	1.643	1	0.120	2.277 (0.818–6.335)^¥^
*Father's occupation*					
Farming (327)	35 (10.70)	0.116	1	0.733	0.889 (0.543–1.457)
Trading (228)	21 (9.21)	1.210	1	0.271	0.714 (0.417–1.223)
Civil servant (31)	3 (9.68)	0.000	1	1.000	0.837 (0.248–2.826)
Daily labourer (30)	10 (33.3)	13.105	1	<0.001^∗^	4.418 (1.977–9.872)^¥^
Others (14)	2 (14.29)	0.000	1	1.000	1.321 (0.290–6.028)
*Mother's occupation*					
Civil servant (343)	22 (6.41)	16.704	1	<0.001^∗^	0.333 (0.196–0.566)
Farming (258)	39 (15.12)	5.830	1	0.016^∗^	1.892 (1.151–3.111)^¥^
Trading (21)	5 (23.81)	2.242	1	0.134	2.571 (0.912–7.246)
Housewife (2)	1 (50.00)	0.378	1	0.539	7.971 (0.493–128.875)
Others (6)	4 (66.67)	13.418	1	<0.001^∗^	16.627 (2.989–92.504)^¥^
*Father's educational status*					
Primary (175)	34 (19.43)	15.020	1	<0.001^∗^	2.724 (1.647–4.506)^¥^
Secondary (340)	20 (5.88)	20.285	1	<0.001^∗^	0.293 (0.170–0.504)
Tertiary (100)	5 (5.00)	3.957	1	0.047^∗^	0.370 (0.145–0.943)
No formal education (15)	12 (80.00)	65.718	1	<0.001^∗^	37.695 (10.342–137.394)^¥^
*Mother's educational status*					
Primary (293)	25 (8.53)	3.609	1	0.058	0.590 (0.353–0.987)
Secondary (292)	28 (9.59)	1.240	1	0.265	0.728 (0.440–1.205)
Tertiary (27)	5 (18.52)	0.823	1	0.365	1.849 (0.678–5.047)
No formal education (18)	13 (72.22)	62.710	1	<0.001^∗^	24.835 (8.550–72.135)^¥^
*Walk barefoot*					
Yes (18)	12 (66.67)	51.305	1	<0.001^∗^	18.746 (6.786–51.783)^¥^
No (612)	59 (9.64)	51.305	1	<0.001^∗^	0.053 (0.019–0.147)
*Wash fruits and vegetables*					
Yes (347)	26 (7.49)	10.120	1	0.001^∗^	0.428 (0.257–0.714)
No (283)	45 (15.90)	10.120	1	0.001^∗^	2.334 (1.400–3.892)^¥^
*Wash hands after using toilet*					
Yes (371)	39 (10.51)	0.350	1	0.554	0.833 (0.507–1.370)
No (259)	32 (12.36)	0.350	1	0.554	1.200 (0.730–1.973)
*Eat soils*					
Yes (366)	55 (15.03)	11.451	1	<0.001^∗^	2.741 (1.533–4.902)^¥^
No (264)	16 (6.06)	11.451	1	<0.001^∗^	0.365 (0.204–0.652)
*Type of house*					
Block (491)	35 (7.13)	36.316	1	<0.001^∗^	0.220 (0.132–0.367)
Mud (139)	36 (25.90)	36.316	1	<0.001^∗^	4.554 (2.729–7.599)^¥^
*Source of drinking water*					
Borehole (101)	10 (9.90)	0.092	1	0.762	0.843 (0.416–1.707)
Stream/river (19)	18 (94.74)	128.017	1	<0.001^∗^	189.509 (24.807–1447.740)^¥^
Well (312)	34 (10.90)	0.028	1	0.868	0.929 (0.567–1.523)
Tap water (198)	9 (4.55)	12.095	1	<0.001^∗^	0.2842 (0.138–0.584)
*Where do you defecate*					
Water closet (388)	20 (5.16)	9.145	1	0.003^∗^	0.426 (0.247–0.733)
Pit latrine (142)	30 (21.13)	16.562	1	<0.001^∗^	2.920 (1.746–4.885)^¥^
Open defecation (100)	21 (21.00)	10.127	1	0.002^∗^	2.552 (1.454–4.479)^¥^
*Treatment for worm*					
Yes (567)	28 (4.94)	221.021	1	<0.001^∗^	0.024 (0.013–0.046)
No (63)	43 (68.25)	221.021	1	<0.001^∗^	41.388 (21.552–79.477)^¥^

^¥^Significantly associated with STH infection; *n*: number examined; ^∗^significant at *p* < 0.05.

## Data Availability

The data sets in this study are available from the corresponding author on reasonable request.

## Abbreviations

STHs: Soil-transmitted helminths
STH: Soil-transmitted helminthiasis
NTDs: Neglected tropical diseases
LGA: Local Government Area
SBD: School-based deworming
NGOs: Nongovernmental organizations
OR: Odds ratio
SPSS: Statistical Package for the Social Sciences
CI: Confidence interval.
